# 735 Early Treatment with NSAIDs Improves Blood Clotting Function in Severely Burned Patients

**DOI:** 10.1093/jbcr/irac012.288

**Published:** 2022-03-23

**Authors:** Lyndon G Huang, Juquan Song, Kassandra K Corona, Kendall Wermine, Elvia L Villarreal, Phillip H Keys, Giovanna De La Tejera, Sunny Gotewal, Alejandro A Joglar, Tsola A Efejuku, Shivan N Chokshi, Shelby P Bagby, Jasmine M Chaij, Maria Haseem, Amina El Ayadi, George Golovko, Steven E Wolf

**Affiliations:** University of Texas Medical Branch, Galveston, Texas; University of Texas Medical Branch, Galveston, Texas; University of Texas Medical Branch, Galveston, Texas; University of Texas Medical Branch, Galveston, Texas; University of Texas Medical Branch, Galveston, Texas; University of Texas Medical Branch, Galveston, Texas; University of Texas Medical Branch, Galveston, Texas; University of Texas Medical Branch, Galveston, Texas; University of Texas Medical Branch, Galveston, Texas; University of Texas Medical Branch, Galveston, Texas; University of Texas Medical Branch at Galveston, Galveston, Texas; University of Texas Medical Branch, Galveston, Texas; University of Texas Medical Branch, Galveston, Texas; University of Texas Medical Branch, Galveston, Texas; University of Texas Medical Branch at Galveston, Galveston, Texas; University of Texas Medical Branch at Galveston, Galveston, Texas; University of Texas Medical Branch at Galveston, Galveston, Texas

## Abstract

**Introduction:**

The risk of coagulopathy is increased in severe burns. Nonsteroidal anti-inflammatory drugs (NSAIDs) are commonly prescribed in burn patients to relieve pain and reduce inflammation. This study investigates the impact of NSAIDs on burn induced coagulopathy in severely burned patients.

**Methods:**

Severe burn patients (total body surface area [TBSA] >20%) were identified with TriNetX, a North American federated health research network from 51 health-care organizations (HCOs) and categorized for those receiving NSAIDs during the first week following injury; those with NSAID use prior to injury were excluded. NSAIDs included in this study were ibuprofen, oxaprozin, indomethacin, aspirin, diclofenac, celecoxib, and naproxen. Burn induced coagulopathy was defined as international normalized ratio (INR) levels ≥1.5. Statistical significance of the rate of burn-induced coagulopathy in the week following injury among the two groups was analyzed with measures of association using chi-squared tests.

**Results:**

We identified 709 severely burned patients receiving NSAIDS during the week after burn and 1,032 severely burned patients without NSAID use. Among those receiving NSAIDs, ibuprofen and aspirin were the most prescribed at rates of 80% and 36%, respectively. After cohort matching, the risk of burn induced coagulopathy was significantly decreased in patients taking NSAIDs (17.7%) compared to patients not receiving NSAIDs (32.3%) (p< 0.0001). The protective nature of NSAIDs was greatest on the same day (p=0.0002) and first day following burn injury (p=0.0026). On average, those not taking NSAIDs had an elevated risk of developing coagulopathy compared to those who did as %TBSA increased in 10% intervals. This observation was confirmed in a linear regression analysis with slopes of 0.0453 and 0.0293, respectively. Furthermore, patients taking NSAIDs were less likely to develop sepsis (p=0.0046) and thrombocytopenia (p=0.0003) and die the first week following injury (p< 0.0001).

**Conclusions:**

The early protective effects of NSAIDs at reducing the risk of coagulopathy occurs during the acute phase of burns, though selection bias cannot be excluded. The potential risk of burn induced coagulopathy increased more with %TBSA in patients without NASIDs.

